# Sustainable maize intensification through site-specific nutrient management advice: Experimental evidence from Nigeria

**DOI:** 10.1016/j.foodpol.2023.102546

**Published:** 2023-11

**Authors:** Miet Maertens, Oyakhilomen Oyinbo, Tahirou Abdoulaye, Jordan Chamberlin

**Affiliations:** a Division of Bio-economics, Department of Earth & Environmental Sciences, KU Leuven, Belgium; b Department of Economics, Swedish University of Agricultural Sciences, Sweden; c International Institute of Tropical Agriculture, Bamako, Mali; d International Maize and Wheat Improvement Center, Nairobi, Kenya

**Keywords:** Technology adoption, Agricultural extension, Green revolution, Fertilizer, Greenhouse gas emission, Randomized controlled trial

## Abstract

There is growing evidence on the impacts of site-specific nutrient management (SSNM) from Asia. The evidence for Sub-Saharan Africa (SSA), where SSNM developments are more recent and where conditions concerning soil fertility and fertilizer use differ importantly from those in Asia, is extremely scarce. We evaluate a SSNM advisory tool that allows extension agents to generate fertilizer recommendations tailored to the specific situation of an individual farmer’s field, using a three-year randomized controlled trial with 792 smallholder farmers in the maize belt of northern Nigeria. Two treatment arms were implemented: T1 and T2 both provide SSNM information on nutrient use and management, but T2 provides additional information on maize price distributions and the associated variability of expected returns to fertilizer use. We estimate average and heterogenous intent-to-treat effects on agronomic, economic and environmental plot-level outcomes. We find that T1 and T2 lead to substantial increases (up to 116%) in the adoption of good fertilizer management practices and T2 leads to incremental increases (up to 18%) in nutrient application rates, yields and revenues. Both treatments improve low levels of nutrient use efficiency and reduce high levels of greenhouse gas emission intensity, after two years of treatment. Our findings underscore the possibility of a more gradual and sustainable intensification of smallholder agriculture in SSA, as compared with the Asian Green Revolution, through increased fertilizer use accompanied by improved fertilizer management.

## Highlights

• Site-specific nutrient management extension advice through digital support tool.
• Three-year randomized control trial with 792 smallholder maize farmers in Nigeria.
• Incremental increases (up to 18%) in nutrient application, yield and revenue.
• Improved nutrient use efficiency and reduced greenhouse gas emission intensity.
• Gradual and sustainable intensification of smallholder agriculture in Africa.

## 1 Introduction

Global demand for crops is estimated to double by 2050 from its 2005–2010 level, with the largest increase expected in Sub-Saharan Africa (SSA) due to both population and income growth (Tilman et al., 2011). Despite rapid agricultural growth in SSA since 2000, meeting its growing food demand will require substantial output growth (Jayne and Sanchez, 2021, Otsuka and Muraoka, 2017). This output growth will have to originate from agricultural intensification and yield increases as possibilities for acreage expansion are limited and continued soil mining has already severely degraded soils in various SSA countries. Increasing fertilizer use as well as improving crop response to mineral fertilizer are widely regarded as essential for agricultural growth in SSA (Vanlauwe and Dobermann, 2020, Jayne and Sanchez, 2021). At the same time, agricultural production and land use account for about 71% of the greenhouse gas (GHG) emissions of the world food system, which itself accounts for 34% of total annual GHG emissions in the world (Crippa et al., 2022). Fertilizers are an important source of agricultural GHG emissions, through the industrial production of mineral fertilizers as well as the farm-gate emissions of nitrous oxide (N_2_O)^1^ (Venterea et al., 2012, Gerber et al., 2016, van Loon et al., 2019). The Green Revolution process in Asia has been associated with rapid expansion of nitrogen-based fertilizers, large nutrient surpluses and associated GHG emissions, especially N_2_O (Graham et al., 2017, Albanito et al., 2021). This raises important policy questions on whether, with current knowledge, the process of agricultural intensification in SSA can be more sustainable than it has been elsewhere in the world (Godfray, 2015, Jayne et al., 2019, Pingali, 2012, Rockström et al., 2017).

In this paper, we assess whether and to what extent site-specific nutrient management (SSNM) advice can contribute to sustainable agricultural intensification in SSA. SSNM advice entails new extension approaches, supported by digital tools, that account for spatial heterogeneity in agronomic conditions and provide site-specific recommendations on soil nutrient management that are tailored to a specific farmer’s field. Traditional agricultural extension systems typically provide blanket or one-size-fits-all fertilizer recommendations in large areas where soil and climate conditions vary across sites (Shehu et al., 2018, Theriault et al., 2018, Burke et al., 2019). Such blanket fertilizer recommendations, even in combination with fertilizer subsidies, have not been very successful in triggering increased fertilizer use and agricultural intensification in SSA – and have led to the overuse of mineral fertilizer in many parts of Asia. A poor fit between fertilizer recommendations and local soil nutrients can result in a poor yield response to fertilizer use, and may thereby disincentivize fertilizer investments by farmers, slowing the agricultural intensification process (Vanlauwe et al., 2015, Rurinda et al., 2020). The inappropriate use of fertilizer contributes to explaining low yield response, low fertilizer usage, and widening gaps in staple crop yields between SSA and the rest of the world (Barrett and Bevis, 2015, Dobermann et al., 2022, ten Berge et al., 2019, Tittonell and Giller, 2013, van Ittersum et al., 2016, van Rooyen et al., 2021). While there is growing evidence on the impact of SSNM from Asian countries, as recently reviewed by Chivenge and co-authors (2021), the evidence base for SSA, where SSNM developments are more recent and where conditions differ importantly from those in Asia, is extremely thin. Some studies point to promising yield and revenue effects in on-farm researcher-managed SSNM trails in Africa (Balemi and Rurinda, 2020, Chivenge et al., 2022, Saito et al., 2015) but studies on the effects of SSNM information provided to farmers under real-world conditions are scarce. We could identify only three such studies, documenting that SSNM advice results in improvements in yields and net revenues for farmers, but not necessarily in increased fertilizer use, in the rice sector (Arouna et al., 2021) and the maize sectors of Nigeria (Oyinbo et al., 2022) and Ethiopia (Ayalew et al., 2022).

We investigate the impact of SSNM extension advice, through the Nutrient Expert tool, on the intensification of smallholder maize production in Nigeria and on economic and environmental outcomes at farm-level. The Nutrient Expert tool is a tablet- or smartphone-based decision support tool that allows extension agents to generate fertilizer recommendations tailored to the specific situation of an individual farmer’s field (Oyinbo et al., 2022, Pampolino et al., 2012). We use a three-year randomized controlled trial (RCT) among 792 households in the maize belt of northern Nigeria. The RCT includes two treatment groups of farmers: T1 farmers who are exposed to SSNM information interventions on nutrient application rates and fertilizer management, and T2 farmers who are exposed to the same SSNM information and additional information on the variability of expected returns to fertilizer investment under different price scenarios. The RCT includes a control group of farmers who receive blanket fertilizer recommendations that are commonly used throughout Nigeria. We estimate intent-to-treat (ITT) effects on various outcome indicators, including fertilizer management practices, nutrient application rates, nutrient use efficiency, maize yields, net revenue, GHG emission per ha and GHG emission intensity (emission per ton maize output), using regression specifications and the Intergovernmental Panel on Climate Change Tier one method to calculate GHG emission in kilogram carbon dioxide equivalent (kg CO_2_ eq.). We additionally analyze heterogeneous ITT effects through quantile regressions. This study contributes to the scarce evidence on impacts of SSNM extension advice for smallholder farmers in SSA, and is the first study to integrate agronomic, economic and environmental impact dimensions. Our study thereby makes an important contribution towards understanding the potential of SSNM towards sustainable agricultural intensification in SSA.

## 2 Concepts and literature review

SSNM first emerged in Asia in the 1990 s, and spread to SSA where it is being developed since the 2000 s for rice and since the 2010 s for maize and cassava^2^ (Chivenge et al., 2021). SSNM provides nutrient management advice that is geared towards a specific farmer’s field by providing the farmer information on the right rates of application of different nutrients (the right balance of N, P_2_O_5_ and K_2_O) to obtain a certain yield, the appropriate sources of (mineral and organic) fertilizer to obtain these nutrient rates, the right placement of fertilizer (e.g., spot application), and the right timing of fertilizer application (e.g., split application, application at sowing) – which is sometimes referred to as the 4R principles of fertilizer use (Dobermann et al., 2022, Johnston and Bruulsema, 2014, Pampolino et al., 2012, Singh, 2019). The rationale of SSNM advice is to improve the yield response to fertilizer use – and thereby improve the nutrient use efficiency (NuUE), defined as the amount of output per unit of nutrients applied – and avoid overuse of fertilizers by improving fertilizer management practices and the nutrient balance on a field.^3^ SSNM development includes the exploitation of data from field trials and crop growth models to develop digital tools for the delivery of extension advice that is specific to a particular plot within a given geographic area.

There is a growing body of evidence on the impact of SSNM advice, with most of the evidence from various Asian countries. A recent review study by Chivenge and co-authors (2021) identifies 61 on-farm trial studies from 11 countries on SSNM impacts, the large majority from Asia (58 studies and 9 countries), and estimates in a *meta*-analysis that on average SSNM reduces fertilizer application rates by 10%, increases yields by 12%, improves revenues by 15%, and improves the agronomic nitrogen use efficiency (NUE) by 40%. In addition, SSNM has been estimated to reduce N_2_O emissions per kg of output by 22% in irrigated rice production in Vietnam and the Philippines (Pampolino et al., 2007), and to reduce GHG emissions (in CO_2_ equivalent) per ha with 2.5% in rice production and with 12 to 20% in wheat production in India (Sapkota et al., 2021). The evidence from Asia suggests that SSNM can contribute to more sustainable agricultural intensification by decreasing fertilizer (over)use, reducing nutrient surpluses, improving NuUE and decreasing GHG emissions per ha stemming from industrial production of fertilizer as well as from N_2_O emissions from fertilizer use (van Loon et al., 2019). Important to understand is that many Asian countries, including China and India, after decades of input intensification during the Green Revolution, are facing large, even excessive, nutrient surpluses, low levels of NuUE and high levels of GHG emissions per ha (Dobermann et al., 2022). In such a context, a rapidly decreasing sustainability trade-off between crop output and economic returns on the one hand and GHG emission intensity of crop production on the other hand can be expected when SSNM improves yields at lower fertilizer levels.

Conditions are different in SSA, with many countries facing nutrient deficits, especially nitrogen (N) deficits, and low crop yields (Dobermann et al., 2022). Fertilizer application rates and the yield response to fertilizer use remain low in many SSA countries, and promoting fertilizer (as well as other Green Revolution technologies) has been more difficult in SSA than elsewhere. Conceptually, the introduction of SSNM in this context, may, by improving the yield response to fertilizer and fertilizer productivity, incentivize farmers to increase fertilizer application rates, thereby accelerating (or initiating) agricultural intensification. SSNM may alter the intensification path by avoiding nutrient surpluses, increasing NuUE and reducing GHG emission per ha. This raises the following policy-relevant questions. Is SSNM likely to result in (rapid) increases in fertilizer use and yields that are associated with reductions in NuUE and fertilizer productivity, increased GHG emissions per ha, and thereby accelerate agricultural intensification in SSA? Or is SSNM likely to result in yield improvements at higher NuUE and fertilizer productivity levels and lower GHG emission levels, thereby making agricultural intensification more sustainable in SSA than it has been elsewhere in the world?

Given that the development of SSNM decision tools for specific crops is more recent in SSA, empirical evidence on SSNM impacts in SSA is much more limited than for Asian countries. We could identify only three studies that analyze the farm-level impact of the delivery of SSNM extension advice to farmers in SSA, all using RCT study designs. Evidence from these studies indicates that SSNM extension advice increases yields with on average 15% for maize production in Ethiopia, with 9 to 18% for maize production in Nigeria, and with 7 to 20% for rice production in Nigeria, depending on whether the SSNM extension treatment is complemented with additional price risk information, insurance or input subsidies (Arouna et al., 2021, Ayalew et al., 2022, Oyinbo et al., 2022). Net farm revenues are also observed to increase by 14 to 23% across the treatments in these studies, although Oyinbo et al. (2022) do not observe a significant revenue increase from a SSNM extension treatment without price risk information. SSNM advice is observed to increase fertilizer use by 15 to 17% for maize production in Nigeria and Ethiopia, respectively (Ayalew et al., 2022, Oyinbo et al., 2022), implying that SSNM is associated with intensification – yet increases are small and gradual, and do not emerge from all treatments in these studies. In addition, for the rice sector in Nigeria, no significant changes are observed in overall fertilizer use, although the type of fertilizer changes with SSNM advice. Importantly, however, none of these studies addresses environmental outcomes, such that no clear conclusions can be drawn on the contributions of SSNM to sustainable agricultural intensification in SSA from the current literature, and no unambiguous policy advice formulated towards the further development and rollout of SSNM as a sustainability tool.

## 3 Background and methods

### 3.1 Study sites and nutrient Expert tool

**Fig. A1:**
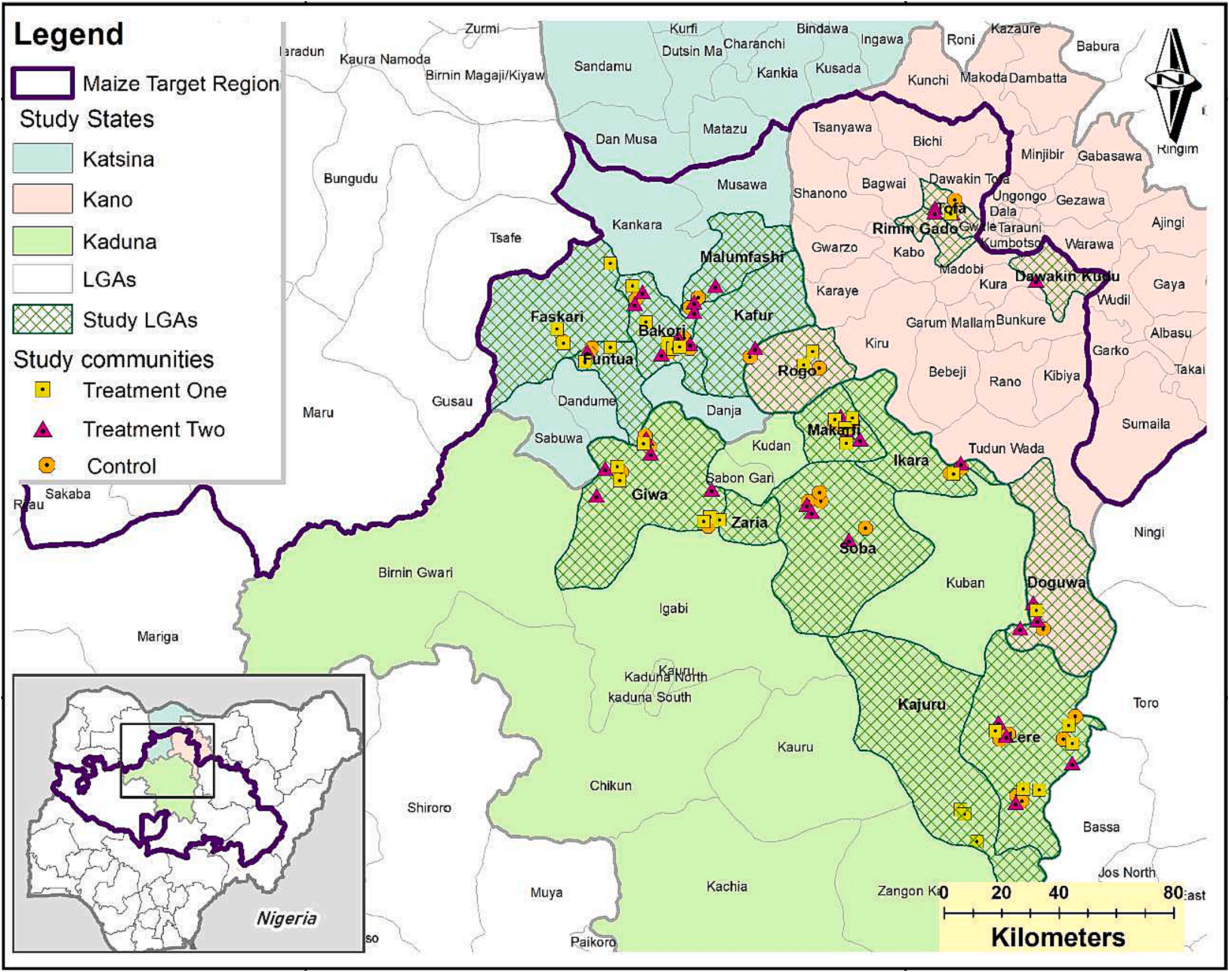
Map of the research area with treatment and control villages.

The research was implemented in Kaduna, Katsina and Kano states in northern Nigeria, covering the northern Guinea, southern Guinea and Sudan Savanna agro-ecological zones (Fig. A1, Appendix A). This region is characterized by a smallholder rainfed maize system with relatively low average fertilizer inputs of about 40 to 50 kg N per ha, and yields of about 1 to 2 ton per ha, which are well below the yield potential of over 5 ton per ha (Liverpool-Tasie et al., 2020, Shehu et al., 2018, ten Berge et al., 2019). While fertilizer application rates are generally higher for maize than for other cereals in Nigeria, they are far below the generally recommended rate of 120 kg N per ha in the study region, and the yield response to applied N is observed to be lower than in other parts of SSA (Liverpool-Tasie et al., 2020). Despite heterogeneous growing conditions in the research area, the current extension system promotes a spatially homogenous fertilizer recommendation of 120 kg N, 60 kg P_2_O_5_ and 60 kg K_2_O per ha of maize (Shehu et al., 2018).

Within this context, the project ‘Taking Maize Agronomy to Scale in Africa (TAMASA)’ co-developed a locally calibrated version of a decision support tool to provide SSNM recommendations to smallholder maize farmers. The tablet- or smartphone-based Nutrient Expert tool^4^ allows extension agents to generate fertilizer recommendations that are tailored to the specific situation of an individual farmer’s field (Pampolino et al., 2012, Oyinbo et al., 2022). The tool is based on the 4R principles of nutrient management – the right fertilizer source, the right fertilizer rate, the right placement and the right time of application (Pampolino et al., 2012, Johnston and Bruulsema, 2014) – and tailors fertilizer recommendations to crop-, plot- and season-specific conditions. The tool uses information about an individual farmer’s plot, input–output price data, and applies the calibrated Quantitative Evaluation of the Fertility of Tropical Soils (QUEFTS) model to derive site-specific nutrient requirements and associated expected agronomic and economic returns (Janssen et al., 1990, Pampolino et al., 2012). The model was calibrated for the study area using data from nutrient omission trials carried out in farmers’ fields in the 2015 and 2016 cropping seasons across diverse soil and climatic conditions in the maize belt of Nigeria (Shehu et al., 2018, Rurinda et al., 2020). The Nutrient Expert tool generates recommendations that include plot-specific information on optimal nutrient rates and fertilizer sources that supply these nutrients as well as general advice on nutrient management practices. The latter is not plot-specific and includes advice on the timing of fertilizer application – in particular on splitting the N application to match nutrient demands at different stages in the maize growth cycle, and including N application at sowing time – and on the fertilizer application method – in particular, spot application is recommended as this reduces nutrient losses and ensures optimal nutrient uptake by the plant.

### 3.2 Sampling and experimental design

A two-stage spatial sampling design was used. In the first stage, 99 villages were randomly selected in the three states^5^ by generating 22 sampling grids of 10 by 10 km across the primary maize-producing areas to ensure spatial representativeness. In the second stage, eight households were randomly selected from a constructed sampling frame of maize-producing farm-households in each of the 99 villages, resulting in a sample of 792 households. We randomly assigned the 99 sampled villages to one control (C) and two treatment groups (T1 and T2, described below), resulting in sub-samples of 264 households from 33 villages in each group.^6^ For each household, the maize plot perceived to be most important for food security or income generation was identified, and all treatment interventions were provided for this focal plot. The sample was designed based on a power calculation with maize yield as the main outcome – with mean 2,232 kg/ha and standard deviation 1,675 derived from the 2015/16 round of LSMS-ISA data – and a 25% yield increase effect. A power of 80% and a significance level of 5%, combined with a conservative intra-cluster correlation coefficient of 0.05, requires a minimum sample size of 61 villages and 488 households for one control-treatment comparison, implying a minimum sample size of 31 villages and 248 households in each group. So the actual sample size, with 33 villages and 264 households per group, slightly exceeds the minimum for a power of 80%.

Farmers in T1 were exposed to SSNM information that is specific to their focal maize plot, including a plot-specific fertilizer application rate to obtain a target yield, optimal fertilizer management practices (sources, timing and placement), the rationale behind the recommendations and a detailed explanation of how to implement them as well as the expected return from the uptake of the recommendations. The latter is estimated based on the prevailing maize market price at the time of providing the information, before planting. This is similar to most agronomic recommendations and is in line with the risk that farmers face due to the time lag between planting decisions and post-harvest output prices. Farmers in T2 were exposed to the same treatment as T1 farmers but received additional information on the variability of expected returns, stemming from maize price fluctuations. This is estimated based on the 25th, 50th and 75th percentiles of the distribution of the monthly real maize price during post-harvest months over the last nine years in the research area.^7^ This additional information on the variability of expected returns is provided to reduce farmers’ information uncertainty on price risks and returns to fertilizer investment – which does not imply that the price risk itself is reduced or the distribution of the return to fertilizer itself is changed. The farmers in C received the general recommendation of 120 kg N, 60 kg P_2_O_5_ and 60 kg K_2_O per ha, which prevails in the agricultural extension systems, and were not exposed to SSNM interventions or associated information on optimal fertilizer management practices and economic returns.

The SSNM information, based on the Nutrient Expert tool, was provided to T1 and T2 farmers by public extension agents prior to planting in the 2017 and 2018 farming seasons (April to May). Extension agents were trained intensively to ensure a proper understanding of how to use the tool, to generate recommendations and to interpret the results to farmers; and supervised in the field to ensure that recommendation protocols were correctly followed. The use of the Nutrient Expert tool requires farmers to provide information on previous crop management practices on the plot (use of inorganic and organic fertilizer, seed type, cropping system, yield, etc.), on characteristics of the growing environment (water availability, incidence of drought, flood, etc.), and on input and maize prices; and extension agents to obtain additional information on soil characteristics (color, texture, etc.) through physical observation and to record the plot location and size by GPS. The output generated by the Nutrient Expert tool includes fertilizer use guidelines (amount, type, timing and placement), crop management practices and a simple profit analysis to compare returns from current and recommended practices. Extension agents explain the details of the output of the Nutrient Expert tool to the farmer and provide a summary of the recommendations in a report sheet in the local language as a reminder for the farmer.

Given that SSNM treatments T1 and T2 result in nutrient recommendations that are on average above the blanket nutrient rates recommended in C (see further Table 2), and that fertilizer application rates among smallholder maize farmers in Nigeria are typically much below the traditional blanket fertilizer recommendations, we hypothesize that access to SSNM in T1 and T2 increases fertilizer use. As the focus in SSNM is on increasing the yield response to fertilizer use through the 4R principles, we expect T1 and T2 to be associated with a higher maize yield and revenue. Given that price risk is an important factor in farmers’ production and technology decisions (Boyd and Bellemare, 2020), we hypothesize that reducing information uncertainty on the variability in expected return to fertilizer investment in T2 can create more rapid increases in fertilizer investment and associated yield and revenue. This hypothesis is consistent with technology adoption models which posit that technology adoption is a process that hinges on farmers’ learning about the risks associated with adoption (e.g., Aldana et al., 2011, Bedi et al., 2022, Conley and Udry, 2010). Farmers update their beliefs about the distribution of the profitability of a certain technology based on their own experience (and also that of their peers). Too high expectations about the returns to a technology might lead to disappointment and dis-adoption of a technology, which is commonly observed in the literature on agricultural technology adoption (Kijima et al., 2011, Lambrecht et al., 2014, Moser and Barrett, 2006). Providing more accurate information about the distribution of the return to fertilizer use in T2 may accelerate farmers’ learning about the profitability of fertilizer use and prevent disappointment effects of SSNM advice, and could therefore lead to a more rapid increase in fertilizer investment. Finally, GHG emissions per ha are expected to increase with increased fertilizer use but GHG emission intensity (per kg of output) might increase or decrease, depending on how strong the effects of SSNM treatments on fertilizer application rates and yields are. If SSNM treatments improve NuUE, GHG emission intensity may reduce, resulting in more sustainable intensification.

### 3.3 Data collection

Three rounds of a farm-household panel survey were conducted during the maize harvest season (September to October): a baseline survey in 2016 and two follow-up surveys in 2017 and 2018. The surveys were implemented using a structured quantitative questionnaire with different modules and plot-, household- and community-level components, computer-assisted personal interviewing software and tablets. An additional community-level survey was done to collect data on input and output prices, access to institutions and services, and incidence of shocks. Survey data include the full sample of 792 maize-producing households at baseline, and 788 and 786 households in 2017 and 2018 respectively, of which 690 and 666 cultivated maize on the focal plot.

Sample attrition is thereby very low: 0.5% in 2017 and 0.8% in 2018. Yet, maize cultivation attrition, which relates to farmers dropping out of maize cultivation on the focal plot, is higher, with attrition rates of 12.5% and 15.5% in 2017 and 2018 respectively. Total attrition has no major impact on the balancing of observable baseline characteristics across treatment and control groups after attrition in 2017 and in 2018 (see Table 1). As common in RCT studies (e.g., Hossain et al., 2019, Arouna et al., 2021), we test for possible differential attrition across treatment groups by regressing a dropout indicator variable against treatment dummies, a time dummy and baseline observable characteristics, and their interaction with treatment dummies. Results (Table A1, Appendix A) show that T1 farmers, compared to C farmers, are significantly less likely to drop out in 2017. Yet, the joint significance of baseline control variables and their interactions with treatment dummies can be rejected, which implies that the potential of differential sample and maize cultivation attrition is likely minimal. We nevertheless test the robustness of the results against potential non-random attrition.

**Table 1:** Baseline characteristics and balance between control (C) and treatment (T1 and T2) groups.

	Panel A: 2016–2017						Panel B: 2016–2018					
	C	T1	T2	T1 = C	T2 = C	T1 = T2	C	T1	T2	T1 = C	T2 = C	T1 = T2
*Household characteristics*												
Age of hh head (years)	44.41	44.20	44.23	0.856	0.871	0.984	44.23	44.91	44.35	0.555	0.916	0.628
	(0.77)	(0.79)	(0.78)				(0.80)	(0.84)	(0.81)			
Education of hh head (years)	5.42	5.34	4.93	0.881	0.385	0.462	5.25	5.07	4.93	0.752	0.573	0.800
	(0.41)	(0.39)	(0.40)				(0.40)	(0.40)	(0.41)			
Household size	9.01	8.93	9.87	0.863	0.105	0.086	8.62	8.96	9.71	0.476	0.040	0.185
	(0.31)	(0.34)	(0.44)				(0.29)	(0.36)	(0.44)			
Livestock ownership (TLU) 2	1.73	1.80	2.29	0.751	0.041	0.067	1.93	2.22	2.26	0.514	0.287	0.932
	(0.16)	(0.15)	(0.22)				(0.22)	(0.39)	(0.22)			
Total farm size (ha)	3.00	3.08	3.37	0.800	0.277	0.384	2.99	3.19	3.40	0.515	0.253	0.570
	(0.21)	(0.22)	(0.27)				(0.23)	(0.22)	(0.28)			
Value of assets (1,000 NGN)	475.67	516.46	608.36	0.503	0.096	0.225	471.77	556.8	584.53	0.197	0.145	0.736
	(45.52)	(40.75)	(64.47)				(41.86)	(50.60)	(64.94)			
Household income (1,000 NGN) 3	176.26	182.49	206.50	0.820	0.377	0.420	187.06	174.4	196.97	0.650	0.775	0.454
	(22.70)	(15.90)	(25.46)				(23.14)	(15.69)	(25.90)			
Maize farming experience (years)	19.01	19.14	18.24	0.885	0.431	0.356	19.29	19.72	18.03	0.669	0.200	0.097
	(0.66)	(0.67)	(0.71)				(0.68)	(0.72)	(0.72)			
*Focal plot characteristics*												
Plot size (ha)	0.81	0.84	0.82	0.688	0.898	0.813	0.75	0.87	0.84	0.141	0.342	0.751
	(0.07)	(0.06)	(0.08)				(0.05)	(0.07)	(0.08)			
Plot ownership (dummy)	0.96	0.94	0.97	0.200	0.928	0.165	0.96	0.93	0.97	0.146	0.800	0.090
	(0.01)	(0.02)	(0.01)				(0.01)	(0.02)	(0.01)			
Distance to homestead (minutes)	14.96	14.33	16.05	0.604	0.536	0.277	15.45	14.7	16.11	0.566	0.716	0.399
	(1.00)	(0.70)	(1.44)				(1.04)	(0.78)	(1.49)			
Use organic fertilizer (dummy)	0.80	0.76	0.77	0.396	0.580	0.770	0.8	0.75	0.76	0.212	0.347	0.762
	(0.03)	(0.03)	(0.03)				(0.03)	(0.03)	(0.03)			
Use improved seed (dummy)	0.27	0.27	0.33	0.884	0.218	0.159	0.28	0.27	0.32	0.825	0.334	0.233
	(0.03)	(0.03)	(0.03)				(0.03)	(0.03)	(0.03)			
Use mineral fertilizer (dummy)	0.97	0.96	0.97	0.401	0.647	0.698	0.97	0.96	0.98	0.482	0.365	0.113
	(0.01)	(0.01)	(0.01)				(0.01)	(0.01)	(0.01)			
NPK fertilizer (kg/ha)	127.77	131.83	132.89	0.697	0.627	0.920	124.05	126.44	131.95	0.811	0.451	0.591
	(7.34)	(7.40)	(7.53)				(7.20)	(6.88)	(7.60)			
Urea fertilizer (kg/ha)	86.94	83.35	91.61	0.677	0.612	0.343	88.09	82.39	90.38	0.513	0.804	0.349
	(6.44)	(5.77)	(6.55)				(6.64)	(5.65)	(6.40)			
Joint $\chi^{2}$ test *p*-values 4				0.991	0.603	0.390				0.872	0.648	0.180
Sub-sample size after attrition	220	240	230				221	225	220			

Standard errors reported between parentheses.

^1^*p*-values associated with two-tailed equality of means tests.

2 TLU, tropical livestock units, an index using weights for different livestock types.

3 Per-adult equivalent annual income.

4 *p*-values associated with $\chi^{2}$ test for baseline characteristics being jointly the same across groups.

**Table 2:** Baseline and control group nutrient application rates, nutrient use efficiency, maize yield and GHG emissions, and recommended nutrient rates from extension treatment interventions.

	Panel A: 2016–2017				Panel B: 2016–2018			
	C		T1	T2	C		T1	T2
	2016	2017	2016		2016	2018	2016	
Fertilizer management practices	0.300	0.268	0.302	0.269	0.285	0.267	0.267	0.291
	(0.031)	(0.03)	(0.030)	(0.029)	(0.030)	(0.03)	(0.030)	(0.031)
N rate (kg/ha)	62.19	74.88***	57.77	58.77	62.13	68.05*	59.58	60.03
	(3.22)	(3.18)	(3.10)	(3.09)	(3.19)	(2.45)	(3.33)	(3.33)
Nutrient rate (kg/ha)	102.9	121.6***	92.58	96.4	102.08	115.8**	94.73	99.04
	(4.40)	(5.40)	(4.21)	(4.43)	(4.40)	(3.97)	(4.47)	(5.06)
NUE (kg output/kg N)	66.48	41.91***	63.59	68.82	65.73	39.55***	60.66	69.09
	(4.92)	(2.10)	(4.52)	(4.63)	(4.83)	(66.08)	(4.58)	(4.79)
NuUE (kg output/kg nutrient)	34.02	25.55***	34.80	35.18	34.01	23.38***	32.56	35.61
	(2.13)	(1.217)	(2.21)	(2.13)	(2.31)	(0.898)	(2.16)	(2.20)
Yield (kg maize/ha)	2,118	2,202	2,010	2,085	2,127	2,243**	1,961	2,108
	(64.07)	(58.10)	(59.09)	(62.93)	(61.07)	(51.61)	(60.08)	(64.10)
Net revenue (NGN / ha)	147,342	127,257**	139,906	145,494	148,247	115,888***	132,653	146,525
	(7,398)	(5,555)	(6,886)	(6,937)	(7,082)	(5,231)	(6,949)	(7,261)
GHG emission (kg CO_2_ eq./ha)	828.3	1,012***	790.5	811.9	824.4	935.1***	797.7	819.9
	(34.69)	(43.03)	(33.56)	(37.04)	(34.74)	(32.63)	(35.45)	(38.26)
GHG emission intensity (kg CO_2_ eq./ton maize)	502.5	507.4	516.7	488.2	473.0	437.9	525.6	478.4
	(32.70)	(24.96)	(34.67)	(35.56)	(27.52)	(14.27)	(35.15)	(31.34)
Recommended N application (kg/ha)	120		129.0***	128.6***	120		132.0***	134.9***
	–		(1.50)	(1.29)	–		(1.48)	(1.64)
Recommended nutrient application (kg/ha)	240		241.6	234.8	240		250.9***	247.1**
	–		(4.66)	(4.01)	–		(3.76)	(4.23)
Sub-sample size after attrition	220	220	240	230	221	221	225	220

Standard errors reported between parentheses. Significant mean differences between C 2016 - C 2017, C 2016 - C 2018, C-T1 and C-T2 indicated as 1% ***, 5% ** and 10% * significance levels.

### 3.4 Estimation strategy

We use the regression specification in equation (1) to estimate the intent-to-treat (ITT) effect:

(1) $$y_{\mathrm{ijt}}=\beta_{0}+\beta_{1}{T1}_{\mathrm{ij}}+\beta_{2}{T2}_{\mathrm{ij}}+\beta_{3}{T1}_{\mathrm{ij}}\tau +\beta_{4}{T2}_{\mathrm{ij}}\tau +\beta_{4}\tau +\beta_{6}y_{ij0}+\beta_{7}X_{ij0}+\varepsilon_{\mathrm{ijt}}$$

where $y_{\mathrm{ijt}}$ is an outcome indicator for the focal plot of household i in village j at year t, ${T1}_{\mathrm{ij}}$ and ${T2}_{\mathrm{ij}}$ are binary indicators for T1 and T2 farmers respectively, τ a time dummy variable for the year 2018, $X_{ij0}$ is a vector of baseline control variables, $y_{ij0}$ is the outcome variable in the baseline year 2016, and $\varepsilon_{\mathrm{ijt}}$ is a random error term. The vector $X_{ij0}$ includes the age and education of the household head, household size, the value of assets, total farm size, size of focal maize plot, plot ownership, and plot distance. The error term is clustered at the village level to account for the clustered sample design and for heteroscedasticity.

To capture agronomic, economic and environmental impact dimensions of SSNM extension advice, we focus on the following outcome indicators: 1) fertilizer management practices; 2) N and nutrient applications rates (in kg /ha); 3) N and nutrient use efficiency, expressed as partial factor productivity of applied nutrients^8^ (in kg maize output / kg nutrients); 4) maize yield (in kg / ha); 5) net revenue from maize production (in NGN / ha); 6) GHG emission (in kg CO2 eq. / ha); and 7) GHG emission intensity (in kg CO2 eq. / ton maize output). The first variable is a dummy variable indicating whether the farmer applies at least three out of the following four fertilizer management practices that are recommended in the SSNM advice treatments: combining mineral with organic fertilizer; split fertilizer application; fertilizer application at sowing; and spot application of fertilizers. Given that N is often the most limiting nutrient in SSA agriculture (Shehu et al., 2018, Rurinda et al., 2020), we focus on N and total nutrient application rates, and on N use efficiency (NUE) and total nutrient use efficiency (NuUE).^9^ Net revenue from maize production is calculated based on average maize prices in specific years, obtained from village surveys. The GHG emission variables were calculated based on the Intergovernmental Panel on Climate Change (IPCC) Tier 1 method (IPCC, 2019). It considers the direct emission of N_2_O from fertilizer application and the indirect emission of N_2_O from volatilization, leaching and run-off – as applied in several empirical studies (e.g., Xu et al., 2022; Sapkota et al., 214 & 2021). In addition, we include CO_2_ emissions from the production of fertilizer using emission factors from the International Fertilizer Association – as applied in Van Loon et al. (2019). To aggregate N_2_O and CO_2_ emissions, the N_2_O global warming potential value from the IPCC Six Assessment Report was applied to convert N_2_O emissions to CO_2_ eq. emissions. The N-containing fertilizer blends that are mostly used by farmers in our study area include NPK 15:15:15 (contains 15% N, 15% P_2_O_5_ and 15% K_2_O), NPK 20:10:10 (20% N, 10% P_2_O_5_ and 10% K_2_O) and urea (46% N).

The ITT effects of the two treatment interventions on all outcome variables are estimated by the coefficients $\beta_{1}$ and $\beta_{2}$ for the first period 2016–2017 (panel A), capturing effects after one year of SSNM advice treatment, and by the linear combinations $\beta_{1}+\beta_{3}$ and $\beta_{2}+\beta_{4}$ for the second period 2016–2018 (panel B), capturing effects after two years of treatment (Table 3, Table 4). In addition, we explore possible heterogeneity in ITT effects across the outcome distribution using quantile regressions. This allows us to examine how treatment effects vary with the levels of the outcome indicators, and test how impacts of SSNM advice vary across farmers. We report quantile ITT effects only for panel B (Table 5, Table 6).

**Table 3:** Average ITT effects of SSNM advice on fertilizer management, nutrient application rates and nutrient use efficiency.

	(1)	(2)	(3)	(4)	(5)
	Fertilizer management^1^	N rate (kg/ha)	Nutrient rate (kg/ha)	NUE^2^	NuUE^3^
*Panel A: 2016–2017*					
T1	0.196***	−3.241	−3.873	4.005	1.818
	(0.054)	(4.864)	(8.016)	(2.732)	(1.642)
T2	0.271***	4.883	6.150	−0.882	−0.692
	(0.052)	(4.902)	(8.377)	(2.724)	(1.613)
*Panel B: 2016–2018*					
T1	0.220***	1.406	3.140	0.641	0.253
	(0.040)	(3.683)	(6.537)	(2.150)	(1.381)
T2	0.336***	11.288***	16.239**	−0.687	−0.367
	(0.043)	(4.076)	(6.527)	(2.082)	(1.266)
*Control group mean at baseline*					
Panel A	0.304	63.946	105.77	66.269	34.675
Panel B	0.290	64.162	105.46	65.171	34.061
N	1,356	1,356	1,356	1,298	1,268

Standard errors in parentheses. Significance levels * *p* <.1, ** *p* <.05, *** *p* <.01.

1 Fertilizer management is a binary variable for using at least three out of the following four fertilizer management practices: combining organic and inorganic fertilizers; split fertilizer application during the season; applying fertilizer at sowing time; and spot fertilizer application or dibbling.

2 NUE = N use efficiency.

3 NuUE = Nutrient use efficiency. Estimations for NUE (4) and NuUE (5) include observations for which N rate and nutrient rate are non-zero.

**Table 4:** Average ITT effects of SSNM advice on yield, revenue and GHG emission and emission intensity.

	(1)	(2)	(3)	(4)
	Yield (kg/ha)	Revenue (NGN/ha)	GHG emission (kg CO_2_ eq./ha)	GHG emission intensity (kg CO_2_ eq./ton)
*Panel A: 2016–2017*				
T1	110.671	7,065	−40.288	−48.525
	(81.578)	(8,233)	(64.939)	(33.147)
T2	238.05***	15,129*	58.134	−27.931
	(81.780)	(7,848)	(66.135)	(31.892)
*Panel B: 2016–2018*				
T1	173.11**	6,013	19.073	−24.584
	(74.52)	(5,361)	(50.013)	(18.784)
T2	380.35**	20,515***	147.94***	−20.434
	(74.31)	(5,221)	(53.224)	(18.584)
*Control group mean at baseline*				
Panel A	2,118	147,342	828.33	502.46
Panel B	2,127	148,247	824.42	473.01
N	1,356	1,356	1,356	1,356

Standard errors in parentheses. Significance levels * *p* <.1, ** *p* <.05, *** *p* <.01.

**Table 5:** Heterogenous ITT effects of SSNM advice on nutrient application rates and nutrient use efficiency, panel B (2016–2018).

	(1)	(2)	(3)	(4)
	N rate (kg/ha)	Nutrient rate (kg/ha)	NUE^1^	NuUE^2^
*T1*				
10th percentile	2.746	7.360	1.600	1.315*
	(3.669)	(5.523)	(1.196)	(0.697)
25th percentile	1.360	1.600	1.730	1.258*
	(3.399)	(7.099)	(1.521)	(0.705)
50th percentile	−0.283	6.243	5.197***	1.364
	(4.047)	(7.198)	(1.807)	(1.234)
75th percentile	−3.741	−2.506	0.277	0.500
	(4.995)	(10.296)	(3.155)	(2.258)
90th percentile	−2.441	3.311	−13.605**	−5.137
	(9.050)	(11.569)	(6.825)	(3.899)
*T2*				
10th percentile	8.180*	9.879	0.769	2.206***
	(4.565)	(8.997)	(1.182)	(0.745)
25th percentile	10.630***	13.300**	0.891	1.904***
	(3.783)	(6.684)	(1.320)	(0.680)
50th percentile	13.058***	18.508***	3.122	1.571
	(4.444)	(6.517)	(1.949)	(1.355)
75th percentile	15.211***	24.438*	−3.040	−1.319
	(5.730)	(12.648)	(2.764)	(2.076)
90th percentile	15.552*	24.856**	−19.599***	−9.113***
	(8.507)	(9.727)	(6.397)	(3.194)
N	1,356	1,356	1,298	1,298

Standard errors in parentheses. Significance levels * *p* <.1, ** *p* <.05, *** *p* <.01.

1 NUE = N use efficiency.

2 NuUE = Nutrient use efficiency. Estimations for NUE (3) and NuUE (4) include observations for which N rate and nutrient rate are non-zero.

**Table 6:** Heterogenous ITT effects of SSNM advice on yields, revenue and GHG emission and emission intensity, panel B (2016–2018).

	(1)	(2)	(3)	(4)
	Yield (kg/ha)	Revenue (NGN/ha)	GHG emission (kg CO_2_ eq./ha)	GHG emission intensity (kg CO_2_ eq./ton)
*T1*				
10th percentile	183.28	9,897	63.156	28.376
	(118.84)	(7,742)	(55.325)	(20.631)
25th percentile	190.33**	8,615	20.633	−16.421
	(96.31)	(7,356)	(58.487)	(20.996)
50th percentile	198.26**	5,426	−1.206	−38.528
	(88.59)	(7,836)	(56.427)	(26.079)
75th percentile	275.49***	8,619	−18.185	−47.448*
	(86.67)	(7,816)	(79.062)	(27.347)
90th percentile	266.75*	−5,104	−47.125	−62.677*
	(140.27)	(13,015)	(91.172)	(34.421)
*T2*				
10th percentile	291.55**	31,024***	143.79**	44.262**
	(135.55)	(7,654)	(66.847)	(19.795)
25th percentile	327.75***	27,983***	133.16**	19.119
	(105.42)	(8,716)	(55.124)	(21.280)
50th percentile	472.06***	23,938***	131.59**	−12.825
	(79.66)	(6,787)	(60.007)	(27.989)
75th percentile	440.30***	11,108	222.63**	−56.564**
	(87.70)	(7,339)	(100.89)	(26.390)
90th percentile	389.14***	4,875	172.07*	−81.804***
	(136.18)	(15,280)	(96.590)	(31.683)
N	1,356	1,356	1,356	1,356

Standard errors in parentheses. Significance levels * *p* <.1, ** *p* <.05, *** *p* <.01.

### 3.5 Robustness

As a robustness check, all estimations for all outcome indicators and the two panel periods, are done without including the vector of baseline control variables $X_{ij0}$, thereby relying on balancing between the treatment groups (Table A2, Table A3, Appendix A). In addition, based on the methods reviewed by Molina-Millan and Macours (2021), we test the robustness of our results against non-random attrition in two ways. First, we estimate a Heckman sample selection model^10^ for all outcome variables, using a two-stage consistent estimator with bootstrapped standard errors, and using the vector of covariates $X_{ij0}$ along with additional variables expressing the years of experience with maize cultivation and participation in maize contract-farming in the sample selection equation (Table A4, Table A5, Appendix A). Second, as is often done in recent RCT studies and as proposed by Lee (2009), we estimate Lee bounds as upper and lower treatment-effects bounds (Table A6, Table A7, Appendix A). Finally, we correct *p*-values for multiple hypothesis testing. Our main analysis includes nine outcome indicators and two treatment variables for which we test null hypotheses on the coefficients of interest. Testing multiple hypotheses may increase the probability of one or more false rejections of the null hypothesis (type I error). We adjust the *p-*values for this using the method proposed byList et al. (2019),^11^ relying on bootstrapping to allow for multiple *p-*values to be correlated and avoid any type I error (Table A8, Appendix A).

## 4 Results

### 4.1 Baseline characteristics

Baseline summary statistics indicate that on average farmers are 44 years old, have about 5 years of formal schooling, 19 years of experience in maize farming, households of about 9 members, 3 ha of farmland and 2 tropical livestock units. The maize focal plot is on average 0.9 ha, located about 15 min walking distance from the homestead, mainly (98%) owned by the farmer, likely cultivated with inorganic fertilizer (97%) and organic manure (78%) but less likely cultivated with improved seeds (29%). Randomization is checked by testing equality of means in baseline characteristics between the three groups (Table 1). The *p*-values of the pairwise comparisons show that almost none of the baseline characteristics differs significantly across the groups – only for livestock and asset ownership in panel A and for household size in panel B there are significant differences between T2 and C farmers. Overall, the $\chi^{2}$ tests of joint orthogonality show that we cannot reject the null hypothesis that the baseline observables are orthogonal to the treatment status, and can conclude that the randomization design produced comparable groups in both panels.

**Fig. A2:**
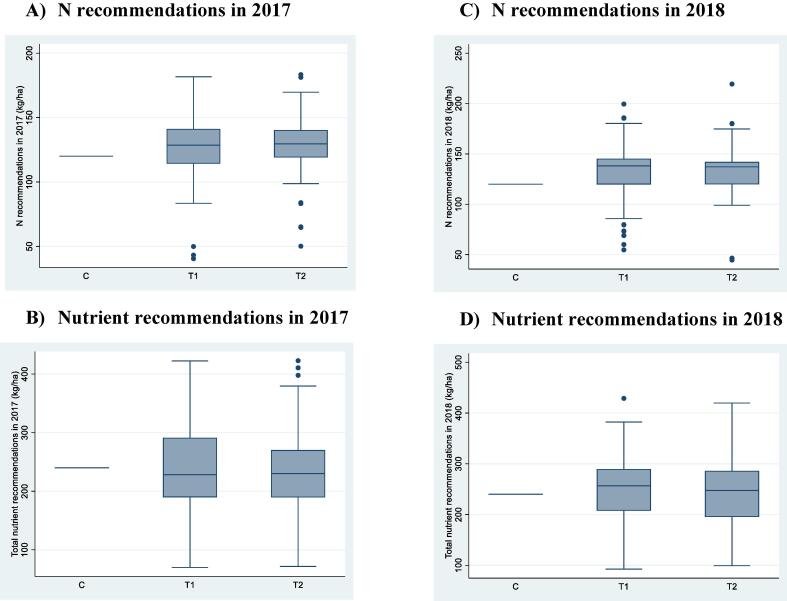
Distribution of recommended N and total nutrient application rates across control (C) treatment (T1 & T2) groups and panel years 2017 & 2018.

The figures in Table 2 show that average baseline N and nutrient application rates, NUE and NuUE, yields, revenue, and GHG emission and emission intensity are similar between treatment and control groups, which stems from randomization. Within the control group, average N and nutrient application rates, yield and GHG emission increase slightly over time while average NUE, NuUE and revenue decrease over time. The average baseline N and total nutrient application rates are well below the general recommendation of 120 kg N per ha and 240 kg of total nutrients per ha. SSNM recommendations for N application in T1 and T2 are on average significantly higher than the general recommendation of 120 kg in C in both panels while total nutrient recommendations from SSNM advice in T1 and T2 are on average significantly higher than the general recommendation of 240 kg nutrients in C only in panel B. In Fig. A2 in the Appendix A, we show that SSNM advice results in recommendations that vary substantially across farmers, and that for about half of the observations in T1 and T2 SSNM recommendations are above the blanket recommendation.

### 4.2 Average ITT effects

Table 3 reports average ITT estimates for the two SSNM treatments, where outcomes of interest are fertilizer management practices, nutrient application rates, and nutrient use efficiency for nutrients in general and N specifically. Results show that both T1 and T2 increase the likelihood of adopting at least 3 out of 4 good fertilizer management practices with 20 to 22 percentage points (pp) for T1 and 27 to 34 pp for T2.^12^ These are substantial effects, implying an increase in the likelihood of good fertilizer management, with respect to the baseline control, of 65 to 76% for T1 and 89 to 116% for T2. Results show that T1 has no significant average effect on N and total nutrient application rates nor on NUE and NuUE, neither after one year nor after two years of SSNM advice. Results show that T2 has significant positive average effects on N and total nutrient application rates after two years, but no significant effects after one year, of SSNM advice. These effects are rather modest with N use increasing on average with about 11 kg per ha or 18% and total nutrient use increasing with about 16 kg per ha or about 15%. The increase in nutrient application associated with T2 in panel B does not result in changes in nutrient use efficiency, as no significant average effects are observed on NUE and NuUE in any of the two years.

Table 4 reports the results of the estimation of the average ITT effects of the two treatments on maize yield, net revenue per ha, and GHG emission and emission intensity. The results show that both T1 and T2 have a positive average effect on yields, T2 in both years and T1 only after two years. These yield effects translate into a significant average effect on net revenue per ha only for T2. Yield and revenue effects are modest with T1 resulting in a yield increase of about 8% and T2 resulting in a yield increase of about 18% and a revenue increase of about 14% after two years of treatment. We find no significant average effects of T1 on GHG emission and emission intensity in both periods, and no significant effect of T2 on these indicators in the first period. We need to note that standard errors are high, and hence estimated ITT effects on GHG emission and emission intensity are imprecise. Yet, we find that T2 on average results in a significant increase in GHG emission per ha but not in emission intensity in the second period. This effect amounts to an average increase in GHG emission of 148 kg CO_2_ eq. per ha or 18%.

The results in Table 3, Table 4 are robust to the exclusion of the control variables (Table A2, Table A3, Appendix A). Point estimates of the ITT effects are very similar in the models with or without control variables, which adds to the credibility of the randomization. In addition, results are largely robust to differential sample and maize cultivation attrition. Point estimates of the ITT effects are qualitatively the same and quantitively very similar to the results from the Heckman selection model (Table A4, Table A5, Appendix A). Lee bounds estimates of significant ITT effects show tight and significant upper and lower treatment-effects bounds that encompass the point estimate (Table A6, Table A7), with some exceptions. Adjusted *p*-values in Table A8 show that significant ITT effects remain significant after adjusting *p-*values for multiple hypothesis testing, with some exceptions. These exceptions relate to the the effects of T2 on yield and revenue in panel A and the effects of T1 on yield in panel B, where lower Lee bounds as well as adjusted *p-*values are not significant, which implies that these effects are less robust.

### 4.3 Heterogenous ITT effects

Table 5 reports the estimates of the quantile treatment effects, after two years of treatment (panel B), for N and nutrient application rates, and NUE and NuUE. We observe no significant effects of T1 on N and total nutrient application rates at different percentiles of the distribution. The effect of T2 on N and total nutrient application rates seems to be higher at higher percentiles of the distribution (and for total nutrients not significant at the 10th percentile), implying that SSNM is more effective in further stimulating fertilizer use among farmers using more fertilizer than among farmers using less fertilizer. Further, results show that treatment effects on NUE and NuUE are positive at lower percentiles and negative at higher percentiles, especially for the effects of T2 on NuUE. These effects suggest that SSNM advice reduces nutrient use efficiency and fertilizer productivity where this is high (at low levels of fertilizer use) but slightly increases nutrient use efficiency and fertilizer productivity where it is low (at higher levels of fertilizer use).

Table 6 reports the estimates of the quantile treatment effects, after two years of treatment (panel B), for yield, net revenue, and GHG emission and emission intensity. We observe significant positive yield effects throughout the yield distribution for both treatments, except at the 10th percentile for T1. The effect of T1 on net revenue is not significant while the effect of T2 on net revenue is significantly positive up to the 50th percentile, and seems to decrease across the distribution. We observe no significant effects of T1 on GHG emission per ha at different percentiles of the distribution, and significant positive effects of T2 on GHG emission per ha at all percentiles. While we observe no significant average ITT effects of the SSNM treatments on GHG emission intensity (Table 4), estimated effects do differ across the distribution. Results point out that SSNM advice, especially T2, increases GHG emission intensity at the lower end of the distribution but reduces GHG emission intensity at the upper end. The effects on GHG emissions are considerable: T2 increases GHG emissions with 133 to 222 kg CO_2_ eq. per ha at the 50% to 75% percentile of the sample (or an increase of 16 to 27% with respect to the baseline control), increases GHG emission with 44 kg per ton maize (or an increase of 9%) at the 10th percentile but reduces GHG emission with 57 to 82 kg per ton maize (or a decrease of 12 to 17%) in the upper percentiles.

## 5 Discussion

We find that SSNM advice, along with information on the distribution of expected returns to fertilizer investment, for maize production in Nigeria increases fertilizer use, yield and net revenue per ha with on average respectively 15%, 18% and 14%, as documented by the estimated average effects of T2 in our analysis. The sizes of these effects are very similar to other estimates of SSNM extension treatment effects for maize and rice production in Nigeria and Ethiopia (Arouna et al., 2021, Ayalew et al., 2022, Oyinbo et al., 2022). The size of these yield and revenue effects are also similar in magnitude to the estimates of an average yield increase of 12% and an average revenue increase of 15% from a *meta*-study on SSNM advice including mainly Asian countries but the effects on fertilizer use are opposite to what is found in Asian countries where SSNM advice decreases fertilizer use with on average 10% across studies (Chivenge et al., 2021), which can be explained by the different context of fertilizer over-use in Asian versus under-use in African countries in general. However, our average yield and revenue effects estimates are lower than what has been reported by studies estimating yield and profitability impacts of SSNM in on-farm researcher-managed trials in Africa (Chivenge et al., 2022), e.g., yield and profit effects of up to 36 and 39% respectively for rice in Senegal (Saito et al., 2015) and yield effects of up to 37% for maize in Ethiopia (Balemi and Rurinda, 2020). These differences in the magnitude of estimated impacts are in line with the systematically lower productivity of technologies as adopted and implemented by farmers under real-world conditions, as compared with researcher-managed trials (Laajaj et al., 2020). This underscores the importance of complementing on-farm trials of SSNM implementations (in comparison to farmers’ traditional practices) with studies on the impacts of actual SSNM extension advice to farmers on their own farms (in comparison to traditional extension advice), in order to more fully understand the potential of SSNM support tools in the real world.

We only find a significant positive average effect of SSNM advice on fertilizer use when SSNM advice is combined with information on the distribution of return to fertilizer investment in T2, and only after two years of treatment. Two aspects of this finding are worth highlighting. First, by parameterizing and explicating risk (in this case, price risk), resource-poor farmers may be more willing to gradually invest in intensification. By avoiding disappointment with realized returns after initial fertilizer investments, information on the variability in returns may contribute to convincing farmers to gradually increase fertilizer investments rather than dis-continue those investments or dis-adopt the technology (Oyinbo et al., 2022) – dis-adoption after trial of a technology is often observed in the agricultural technology adoption literature (e.g. Kijima et al., 2011, Lambrecht et al., 2014). Secondly, however, the effects of T2 are modest with an average increase in nutrient application rates of 16 kg per ha only, and smaller among farmers using less fertilizer. Post-treatment nutrient application is on average still substantially below the recommendations while nutrient application rates also increase in the control group, with on average 14 kg per ha between 2016 and 2018. This implies that SSNM advice is not the silver bullet for spurring agricultural intensification in Africa but rather an approach to accelerate the speed of gradual increases in fertilizer use and yields (Oyinbo et al., 2022). Yet, positive yield and revenue effects are observed after one year of treatment for T2, and positive yield effects after two years of treatment for T1 despite no significant effect on nutrient application rates in these cases. In addition, we observe substantial yield and revenue effects of T2 at the lower end of the distributions, after two years of treatment, despite smaller (and insignificant) effects on fertilizer use. These observations suggest that the treatments in our study may affect yields and revenue primarily through the adoption of improved fertilizer management practices, rather than through increased fertilizer use – or through more balanced nutrient application rather than higher nutrient application rates. This is in line with the large and consistent effect of both treatments on the likelihood to apply the recommended fertilizer management practices, in both years.

This is the first study to estimate GHG emission effects of SSNM extension advice in Africa. Our results point to an average increase in GHG emission per ha of 17% from T2 after two years of treatment while studies on Asia document a GHG emission-reducing effect of SSNM (e.g., Pampolino et al., 2007, Sapkota et al., 2021). In addition, we find that SSNM advice in T2 increases GHG emission intensity at the lower end of the distribution but reduces GHG emission intensity at the upper end. The latter emission intensity-reducing effects are about 12 to 17% and smaller than what is reported in agronomic studies on SSNM in Asian contexts and under researcher-managed trials (Dobermann et al., 2022, Pampolino et al., 2007, Sapkota et al., 2014, Sapkota et al., 2021, Xu et al., 2016, Banayo et al., 2018, Xu et al., 2022). We may also note that GHG emission per ha and GHG emission intensity in our study were much lower at baseline than what is generally observed in Asia. In the context of low fertilizer use in Africa, the primary policy emphasis is on intensifying the use of fertilizer to improve yields and stimulate agricultural growth, and not on reducing GHG emissions from fertilizer use. The finding that SSNM advice can reduce GHG emission intensity in the smallholder maize sector in Nigeria, at relatively low levels of GHG emission intensity, is promising with respect to future sustainable agricultural intensification in Africa.

## 6 Policy implications

Our results on SSNM advice for smallholders in the maize belt of Nigeria document that intensification of smallholder agriculture in SSA is possible. Findings also suggest that this intensification process can be more sustainable in SSA than it has been elsewhere in the world. Yet, even with digitally-enabled SSNM extension advice, progress towards increased fertilizer use and improved yields is only incremental. The findings from the two treatments in this study suggest that reducing farmers’ information uncertainty about the return to fertilizer investment may to some extent accelerate the gradual increase in fertilizer use. Extension systems in general could pay more attention to providing such information in order to avoid disappointment effects and stimulate the gradual intensification process and the continued adoption of agricultural technologies.

Furthermore, our findings suggest that SSNM extension advice can contribute to improving yields, increasing low levels of nutrient use efficiency and decreasing high levels of GHG emissions – and thereby contribute to sustainable intensification in SSA. Yet, our results suggest that these effects are more likely driven by the adoption of good fertilizer management practices than by an increase in nutrient application rates to recommended levels. Given that recommendations on fertilizer management practices are not site-specific – only the recommendations on nutrient application rates are plot-specific – this finding suggests that more effective deployment of non-site-specific advisory tools may also be a useful strategy. Moreover, this would avoid some of the development costs of site-specific recommendation tools. There are no evaluations of the development costs of such tools available yet, and a detailed cost and benefit calculation is also outside the scope of this paper. However, based on our knowledge on the development process for the Nutrient Expert tool for maize in northern Nigeria, we assess that site-specific recommendations based on nutrient-omissions trials involve research and development (R&D) investments in the order of at least one million USD for a new geography. While the per-farm costs of such investments may be reasonable when evaluated against several million potential beneficiary farmers, such costs may be significant barriers to the development of new advisory tools in resource-constrained extension systems or for agro-ecological regions with a small farm population. While this study identifies important potential benefits of further SSNM advisory development, especially in terms of sustainable intensification, the merits of scaling up SSNM extension tools ultimately depend on how SSNM compares to other agricultural R&D expenditures. Nonetheless, SSNM might have an important role to play in helping SSA to avoid going through a similar process of fertilizer overuse and nutrient surpluses as many Asian countries did, especially as input intensity increases, and therefore its further development deserves research attention. At present, however, in the maize belt in northern Nigeria – like other parts of SSA where both nutrient application rates and nutrient use efficiency are similarly very low – improved delivery of non-site-specific extension delivery that focuses on good fertilizer management might be a cost-effective strategy to increase fertilizer use and its efficiency.

## CRediT authorship contribution statement

**Miet Maertens:** Conceptualization, Methodology, Formal analysis, Writing – original draft, Visualization. **Oyakhilomen Oyinbo:** Conceptualization, Methodology, Formal analysis, Investigation, Data curation, Writing – review & editing, Visualization. **Tahirou Abdoulaye:** Conceptualization, Methodology, Investigation. **Jordan Chamberlin:** Conceptualization, Methodology, Writing – review & editing, Project administration, Funding acquisition.

## Declaration of Competing Interest

The authors declare that they have no known competing financial interests or personal relationships that could have appeared to influence the work reported in this paper.

## Figures and Tables

**Table A1:** Differential attrition – results from probit models estimating the probability of attrition.

	Attrition probability	Attrition probability	Attrition probability	Attrition probability
T1	−0.203	−0.368**	−0.362**	−1.476
	(0.129)	(0.154)	(0.152)	(1.378)
T2	−0.071	−0.165	−0.158	0.246
	(0.121)	(0.159)	(0.159)	(1.088)
Panel B		−0.015	−0.010	−0.000
		(0.096)	(0.096)	(0.095)
T1 * Panel B		0.304*	0.297*	0.299*
		(0.160)	(0.160)	(0.158)
T2 * Panel B		0.180	0.175	0.166
		(0.185)	(0.185)	(0.183)
Age of hh head			−0.002	−0.004
			(0.004)	(0.007)
Education of hh head			0.003	0.003
			(0.009)	(0.013)
Household size			−0.004	−0.033**
			(0.010)	(0.016)
Value of assets (log)			0.026	0.023
			(0.034)	(0.048)
Farm size			−0.017	0.008
			(0.016)	(0.026)
Plot size			−0.057	−0.063
			(0.064)	(0.108)
Plot ownership			0.057	0.334
			(0.209)	(0.384)
Distance to homestead			0.001	0.000
			(0.002)	(0.004)
Constant	0.975***	0.967***	1.187**	1.103
	(0.083)	(0.103)	(0.523)	(0.771)
Interactions baseline variables * treatment			No	Yes
*p-*values for joint significance tests				
baseline variables			0.687	0.398
baseline variables * T1				0.215
baseline variables * T2				0.185
N	1,584	1,584	1,584	1,584

Standard errors in parentheses. Significance levels * *p* <.1, ** *p* <.05, *** *p* <.01.

**Table A2:** Average ITT effects of SSNM advice on fertilizer management, nutrient application rates and nutrient use efficiency – results without control variables.

	(1)	(2)	(3)	(4)	(5)
	Fertilizer management	N rate (kg/ha)	Nutrient rate (kg/ha)	NUE	NuUE
*Panel A: 2016–2017*					
T1	0.202***	−3.228	−3.811	4.056	1.851
	(0.054)	(4.851)	(8.057)	(2.760)	(1.657)
T2	0.274***	4.940	6.391	−1.146	−0.852
	(0.053)	(4.717)	(8.079)	(2.728)	(1.608)
*Panel B: 2016–2018*					
T1	0.228***	1.446	3.119	0.528	0.176
	(0.040)	(3.703)	(6.592)	(2.167)	(1.364)
T2	0.342***	11.530***	16.676**	−1.073	−0.617
	(0.044)	(4.067)	(6.489)	(2.116)	(1.289)
*Control group mean at baseline*					
Panel A	0.304	63.946	105.77	66.269	34.675
Panel B	0.290	64.162	105.46	65.171	34.061
N	1,356	1,356	1,356	1,298	1,268

Standard errors in parentheses. Significance levels * *p* <.1, ** *p* <.05, *** *p* <.01. NUE = N use efficiency; NuUE = Nutrient use efficiency. Estimations for NUE (4) and NuUE (5) include observations for which N rate and nutrient rate are non-zero.

**Table A3:** Average ITT effects of SSNM advice on yield, revenue and GHG emission and emission intensity – results without control variables.

	(1)	(2)	(3)	(4)
	Yield (kg/ha)	Revenue (NGN/ha)	GHG emission (kg CO_2_ eq./ha)	GHG emission intensity (kg CO_2_ eq./ton)
*Panel A: 2016–2017*				
T1	109.86	7,259	−40.335	−48.998
	(84.41)	(8,383)	(64.784)	(32.939)
T2	228.00***	14,740*	59.332	−27.097
	(84.09)	(8,039)	(63.551)	(31.229)
*Panel B: 2016–2018*				
T1	170.74**	6,104	19.147	−24.386
	(75.02)	(5,478)	(50.295)	(18.583)
T2	373.14***	20,270***	151.27***	−18.580
	(76.55)	(5,463)	(52.893)	(18.739)
*Control group mean at baseline*				
Panel A	2,118	147,342	828.33	502.46
Panel B	2,127	148,247	824.42	473.01
N	1,356	1,356	1,356	1,356

Standard errors in parentheses. Control group mean at baseline reported. Significance levels * *p* <.1, ** *p* <.05, *** *p* <.01.

**Table A4:** Average ITT effects of SSNM advice on fertilizer management, nutrient application rates and nutrient use efficiency – results from sample selection model.

	Selection model	Outcome models				
		(1)	(2)	(3)	(4)	(5)
		Fertilizer management	N rate (kg/ha)	Nutrient rate (kg/ha)	NUE	NuUE
*Panel A: 2016–2017*						
T1	0.375***	0.143*	−3.865	−5.565	4.230	1.850
	(0.142)	(0.076)	(6.024)	(11.600)	(4.153)	(2.483)
T2	0.155	0.246***	4.586	5.345	−0.776	−0.676
	(0.151)	(0.053)	(4.605)	(9.227)	(2.679)	(1.704)
*Panel B: 2016–2018*						
T1	0.375**	0.207***	1.262	2.752	0.687	1.850
	(0.154)	(0.053)	(4.416)	(7.498)	(2.028)	(2.483)
T2	0.155	0.336***	11.281***	16.220***	−0.676	−0.676
	(0.153)	(0.050)	(3.302)	(6.249)	(2.081)	(1.704)
Maize experience	−0.009*					
	(0.005)					
Maize contract	0.212*					
	(0.113)					
Inverse Mills ratio		−0.440	−5.253	−14.261	1.878	0.273
		(0.313)	(34.096)	(57.398)	(25.304)	(18.136)
N	1,584	1,584	1,584	1,584	1,526	1,526

Standard errors in parentheses. Significance levels * *p* <.1, ** *p* <.05, *** *p* <.01. Baseline covariates included in outcome and selection equations but not reported. NUE = N use efficiency; NuUE = Nutrient use efficiency. Estimations for NUE (4) and NuUE (5) include observations for which N rate and nutrient rate are non-zero.

**Table A5:** Average ITT effects of SSNM advice on yield, revenue and GHG emission and emission intensity – results from sample selection model.

	Selection model	Outcome models			
		(1)	(2)	(3)	(4)
		Yield (kg/ha)	Revenue (NGN/ha)	GHG emission (kg CO_2_ eq./ha)	GHG emission intensity (kg CO_2_ eq./ton)
Panel A: 2016––2017					
T1	0.375***	28.753	6,431	−53.089	−34.887
	(0.142)	(88.608)	(8,899)	(73.016)	(42.264)
T2	0.155	199.05**	14,828*	52.037	−21.446
	(0.151)	(96.850)	(7,587)	(68.715)	(35.518)
Panel B: 2016––2018					
T1	0.375**	154.198*	5,866	16.134	−21.417
	(0.154)	(93.141)	(7,272)	(59.891)	(22.632)
T2	0.155	379.39**	20,507***	147.79**	−20.272
	(0.153)	(98.745)	(6,772)	(65.456)	(20.721)
Maize experience	−0.009*				
	(0.005)				
Maize contract	0.212*				
	(0.113)				
Inverse Mills ratio		−688.96	−5,329	−107.84	114.68
		(658.93)	(50,016)	(414.39)	(217.99)
N	1,584	1,584	1,584	1,584	1,584

Standard errors in parentheses. Significance levels * *p* <.1, ** *p* <.05, *** *p* <.01. Baseline covariates included in outcome and selection equations but not reported.

**Table A6:** Average ITT effects of SSNM advice on yield, revenue and GHG emission and emission intensity – Lee bounds estimates.

		(1)	(2)	(3)	(4)	(5)
		Fertilizer management	N rate (kg/ha)	Nutrient rate (kg/ha)	NUE	NuUE
Panel A: 2016–2017						
T1	Lower bound	0.155***	−12.385***	−20.667**	−3.742	−2.357
		(0.047)	(4.185)	(8.236)	(4.386)	(2.291)
	Upper bound	0.245***	1.697	4.165	6.345*	3.338*
		(0.050)	(4.111)	(7.942)	(3.811)	(1.806)
T2	Lower bound	0.250***	−0.477	−3.727	−5.299	−2.937
		(0.049)	(4.704)	(9.148)	(4.214)	(2.105)
	Upper bound	0.295***	8.217*	11.332	−0.510	−0.142
		(0.046)	(4.703)	(7.963)	(2.688)	(1.929)
Panel B: 2016–2018						
T1	Lower bound	0.217***	−1.392	−1.102	−0.387	0.282
		(0.043)	(3.463)	(6.476)	(2.384)	(1.585)
	Upper bound	0.235***	2.514	4.640	0.909	0.969
		(0.043)	(3.510)	(5.927)	(2.060)	(1.257)
T2	Lower bound	0.341***	11.119***	15.928***	−1.196	−1.571*
		(0.040)	(3.542)	(5.240)	(1.711)	(0.896)
	Upper bound	0.345***	12.596***	17.330**	−0.914	−0.623
		(0.043)	(4.008)	(6.841)	(2.856)	(1.023)

Standard errors in parentheses. Significance levels * *p* <.1, ** *p* <.05, *** *p* <.01. NUE = N use efficiency; NuUE = Nutrient use efficiency. Estimations for NUE (4) and NuUE (5) include observations for which N rate and nutrient rate are non-zero.

**Table A7:** Average ITT effects of SSNM advice on fertilizer management, nutrient application rates and nutrient use efficiency – Lee bounds estimates.

		(1)	(2)	(3)	(4)
		Yield (kg/ha)	Revenue (NGN/ha)	GHG emission (kg CO_2_ eq./ha)	GHG emission intensity (kg CO_2_ eq./ton)
Panel A: 2016––2017					
T1	Lower bound	−87.727	−8,500	−165.499**	−118.96***
		(106.84)	(9,055)	(67.627)	(39.32)
	Upper bound	222.14**	21,441**	28.870	−13.94
		(93.25)	(8,747)	(60.587)	(37.77)
T2	Lower bound	118.50	5,224	−13.815	−65.62
		(114.71)	(10,546)	(59.713)	(41.03)
	Upper bound	300.77***	21,909**	103.821	−6.73
		(104.99)	(9,670)	(67.624)	(36.26)
Panel B: 2016–2018					
T1	Lower bound	105.79	1,696	−17.503	−38.08
		(80.10)	(7,366)	(39.301)	(23.23)
	Upper bound	174.66**	7,780	33.138	−16.93
		(79.95)	(6,072)	(46.538)	(20.18)
T2	Lower bound	363.36***	18,692**	146.61***	−20.54
		(71.30)	(8,641)	(56.02)	(18.09)
	Upper bound	383.14***	21,302***	158.49**	−14.69
		(76.34)	(7,865)	(65.67)	(17.59)

Standard errors in parentheses. Significance levels * *p* <.1, ** *p* <.05, *** *p* <.01.

**Table A8:** Adjusted p-values for multiple hypothesis testing.

		Panel A: 2016 – 2017			Panel B: 2016 – 2017		
Outcome variable	Treatment	Mean diff.	Original *p*-values	Adjusted *p*-values	Mean diff.	Original *p*-values	Adjusted *p*-values
Fertilizer management	T1	0.203	0.000	0.000	0.226	0.000	0.000
	T2	0.271	0.000	0.000	0.342	0.000	0.000
N rate	T1	3.444	0.507	0.982	1.322	0.697	0.943
	T2	4.773	0.297	0.944	11.43	0.006	0.014
Nutrient rate	T1	4.564	0.637	0.974	2.583	0.637	0.934
	T2	5.917	0.431	0.982	16.45	0.012	0.057
NUE	T1	5.150	0.145	0.927	1.400	0.808	0.948
	T2	4.813	0.675	0.954	3.534	0.613	0.975
NuUE	T1	2.633	0.267	0.978	0.393	0.898	0.995
	T2	1.050	0.598	0.948	0.973	0.633	0.978
Yield	T1	93.02	0.196	0.931	144.8	0.025	0.474
	T2	222.8	0.008	0.139	370.2	0.000	0.000
Revenue	T1	6,709	0.389	0.985	4,949	0.268	0.984
	T2	14,60	0.070	0.574	20,14	0.000	0.014
GHG emission	T1	43.74	0.535	0.983	16.74	0.704	0.943
	T2	57.85	0.353	0.968	150.9	0.005	0.013
GHG emission intensity	T1	48.92	0.140	0.777	24.10	0.192	0.917
	T2	27.17	0.388	0.981	18.55	0.324	0.960

Original *p-*values derived from Table A2, Table A3 (ITT estimates without control variables). Adjusted *p-*values estimated following List et al. (2019).

## Footnotes

1 Applied nutrients from fertilizer use that are not taken up by plants run off into waterways or decompose in the soil, thereby releasing N_2_O into the atmosphere.

2 In particular, SSNM for rice is being developed by AfricaRice in the RiceAdvice tool, for maize in the Nutrient Expert tool under the project TAMASA, and for cassava in the AKILIMO tool under the ACAI project.

3 An additional goal of SSNM advice is to improve economic efficiency of fertilizer use through switching to less expensive available fertilizer blends to meet plot nutrient needs, and thereby reduce costs.

4 We refer to Oyinbo and co-authors (2022) for more detailed information on the development and application of the Nutrient Expert tool.

5 The 99 villages are located in 17 Local Government Areas, the administrative unit below the state.

6 We prefer a village-level randomization over a household-level randomization to avoid unintended behavioral and spillover effects that can interfere with causal identification and result in violation of SUTVA (Athey and Imbens, 2017).

7 Price data are derived from weekly nominal maize price data collected from grain markets in the study area by the National Agricultural Extension and Rural Liaison Services (NAERLS), Ahmadu Bello University, Nigeria.

8 NUE and NuUE are sometimes referred to by various other terms in the literature, including partial factor productivity and agronomic efficiency (Congreves et al., 2021). For this paper, we opted for the economic expression of partial factor productivity.

9 For NUE and NuUE, we use 5% winsorized variables to reduce the influence of large outlying values for observations where N and nutrient application rates are very low.

10 We opt for a Heckman model over an inverse probability weighting method because attrition is likely affected by unobservable variables (Molina-Millan and Macours, 2021).

11 The approach of List et al. (2019) is shown to be better able to detect false rejections of the null hypothesis than the more classical Bonferroni and Holm adjustments in the case of multiple treatments.

12 ITT effects for each of the four individual fertilizer management practices show a significant positive effect for all four individual practices for both T1 and T2 and in both panel periods, with estimates ranging from 10 to 24 pp.
